# Management of recently traumatized maxillary central incisors by partial pulpotomy using MTA: Case reports with two-year follow-up

**DOI:** 10.4103/0972-0707.66724

**Published:** 2010

**Authors:** M. Abarajithan, N. Velmurugan, D Kandaswamy

**Affiliations:** Department of Conservative Dentistry and Endodontics, Meenakshiammal Dental College and Hospitals, Chennai, India; 1 Department of Conservative Dentistry and Endodontics, Ramachandra Dental College and Hospitals, Chennai, India

**Keywords:** Partial pulpotomy, mineral trioxide aggregate, vital pulp therapy

## Abstract

In traumatized, young, permanent teeth, pulpotomy is classically undertaken to promote apexogenesis. The objective is to promote root development and apical closure. Once root end development and apical closure is achieved, the root canal treatment is completed. However, it has been suggested that mere pulp exposure does not cause pulpitis in the absence of bacteria. Recent studies have proposed that as long as a good seal is ensured, root canal treatment may not be necessary following pulpotomy. In this article we report two cases of traumatized, fully matured, maxillary permanent central incisors, which have been treated with mineral trioxide aggregate following partial pulpotomy, with a two-year follow-up.

## INTRODUCTION

Preservation of pulp vitality is of paramount importance because, a vital functioning pulp is capable of initiating several defense mechanisms to protect the body from bacterial invasion; it is beneficial to preserve the vitality and health of an exposed pulp rather than to replace it with a root filling material following pulp exposure.[1] Partial pulpotomy is a form of vital pulp therapy that consists of the surgical amputation of 2 – 3 mm of damaged, inflamed, coronal pulp tissue, followed by placing a biocompatible agent to promote healing and maintain vitality of the remaining pulp tissue.[2]

In young permanent teeth, pulpotomy is classically undertaken to promote apexogenesis. The objective is to promote root development and apical closure. Once root end development and apical closure are achieved, the root canal treatment is completed.[2] However, it has been proved that mere pulp exposure does not cause pulpitis in the absence of bacteria.[3] Recent studies have proposed that as long as a hermetic seal is ensured, root canal treatment is not necessary following pulpotomy.[4]

Despite the long history of usage of a calcium hydroxide dressing in different forms, for vital pulp therapy, several disadvantages have been listed with the use of calcium hydroxide material, such as, obliteration of the pulp chamber, high solubility in oral fluids, and lack of adhesion to the dentin.[5–7] There have been attempts to find other suitable materials that will permit dentin bridge formation, as an alternative to calcium hydroxide.[5] The other disadvantage being that multiple visits are required and animal studies have proved that the hard tissue formed under calcium hydroxide has tunnel defects.[6] It is well-documented that sealing the cavities from bacterial ingress is a determinant factor for the success of vital pulp treatment.[4]

Several *in vitro* and *in vivo* studies have reported that MTA has good physical characteristics[8] and is biocompatible.[9] It also provides a very good seal,[10] has excellent marginal adaptation,[11] and maintains a high pH[12] for a long period of time. Of late, Agamy *et al*. concluded that Gray MTA was superior to formocresol and white MTA, when used for pulpotomies in primary teeth.[13]

In this article we report two cases of traumatized maxillary permanent central incisors, which were treated with MTA following partial pulpotomy.

## CASE REPORTS

### Case 1

A 15-year-old male patient reported to our department with a complaint of a fractured upper anterior tooth. History revealed trauma to the tooth 24 hours prior. On examination, an Ellis class III fracture with clinical pulp involvement was seen in relation to 21. The patient experienced pain on stimulus. The exposed pulp (2 × 2 mm) was bright red in color, which was an indication of patent blood supply to the pulp. The tooth responded normally to the electric pulp testing that was performed on the labial surface, which signified the healthy status of the pulp. Hence, a partial pulpotomy procedure was planned with MTA.

### Case 2

A 25-year-old male patient reported to our department with the complaint of a fractured upper anterior tooth. History revealed trauma to the tooth 48 hours prior. On examination, an Ellis class III fracture with clinical pulp involvement was seen in relation to 21. The patient experienced pain on stimulus. The exposed pulp was bright red in color, which was an indication of patent blood supply to the pulp. The tooth responded normally to the electric pulp testing that was performed on the labial surface, which signified the healthy status of the pulp. Hence a partial pulpotomy procedure was planned with MTA.

### Clinical procedure

The tooth was anesthetized with local infiltration of 0.6ml lignocaine (1:200000 adrenaline) and isolated with a rubber dam. The teeth were disinfected with chlorhexidine. The sharp fractured margins were smoothened; the exposed pulp and surrounding dentin were flushed clean with isotonic saline solution. The superficial layer of the exposed pulp and the surrounding tissue were excised to a depth of 2 mm using a high speed diamond bur, with a light touch, using a water coolant. The surface of the remaining pulp was irrigated with isotonic saline until the bleeding was arrested. White MTA was freshly mixed and placed over the exposed pulp, following which a saline-soaked cotton pellet was placed over the MTA for 45 minutes to allow it to set. The exposed dentin and MTA were both sealed with GIC (Glass ionomer cement) and a direct composite restoration was performed, to build up the fractured tooth structure.

### Follow-ups

**Figure 1:**
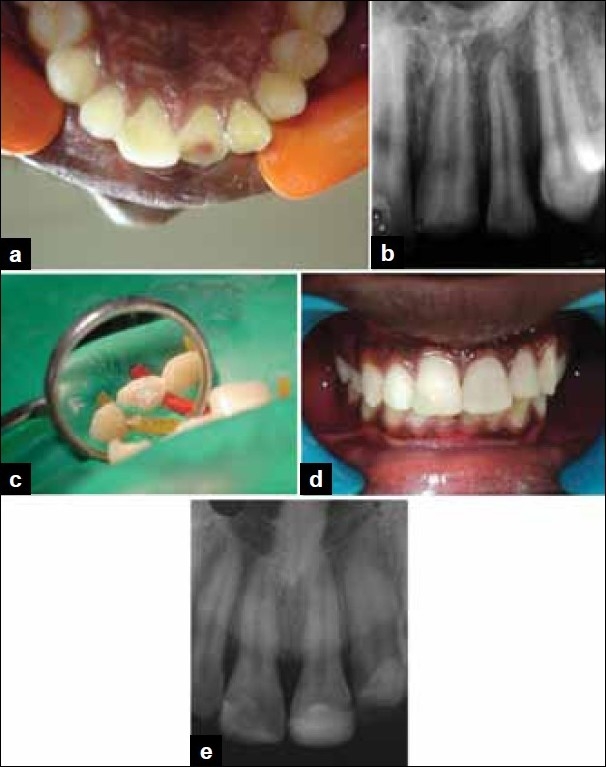
(a) Preoperative photograph showing Ellis class III fracture in 21 with pulp exposure, (b) Preoperative radiograph revealing fracture in 21 with pulp involvement, (c) Partial pulpotomy performed in 21 and MTA placed over the exposed pulp, (d) Postoperative photograph, (e) Postoperative radiograph after two years showing no radiographic changes

**Figure 2:**
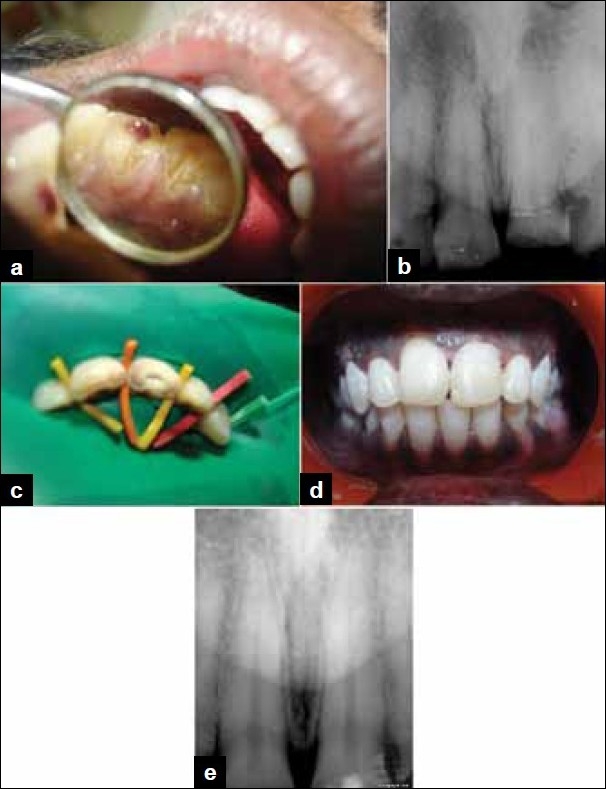
(a) Preoperative photograph showing Ellis class III fracture in 21 with pulp exposure, (b) Preoperative radiograph revealing fracture in 21 with pulp involvement, (c) Partial pulpotomy performed in 21 and MTA placed over the exposed pulp, (d) Postoperative photograph, (e) Postoperative radiograph after two years showing no radiographic changes

Periodic follow-ups were carried out at one, seven, 15, and 30 days, three months, six months, one year, one-and-a-half-years, and two years. Pulp sensitivity was tested with the help of a pulse-oximeter (Custom made pulse-oximeter probe developed by Gopikrishna *et al*.)[14] and an electric pulp tester (EPT) after 24 hours. The tooth was found to respond positively during the follow-up visits. The intraoral periapical radiograph of the tooth was also taken; the periapical region appeared normal (lamina dura was intact without any peripaical changes) [Figures 1 and 2].

## DISCUSSION

Several factors influence the treatment decisions of a clinician when encountering a tooth with a pulp exposure. Cvek *et al*. reported that partial pulpotomies after complicated crown fractures had a 96% success rate.[15] One of the advantages of partial pulpotomy, when compared to cervical or complete pulpotomy, is the preservation of the cell-rich coronal pulp tissue. This tissue possesses better healing potential and can maintain the physiological deposition of dentin.[16] However, the chances of the pulp becoming necrotic or developing a calcific metamorphosis are also present, and hence, a periodic follow-up is mandatory in such cases. However, age is a very important criterion for the selection of patients for any vital pulp therapy. Older pulps are more fibrous, less cellular, and may have less blood supply, thus affecting the treatment outcome.[17]

Studies on animal models have proved that the hard tissue bridge under calcium hydroxide has many imperfections and tunnel defects that may permit bacterial leakage, whereas, the bridge formed after MTA placement is complete with no tunnels or imperfections. Moreover, in human teeth it is reported that at six months, a 0.43 mm thick dentin bridge is evident when MTA is used compared to 0.15 mm with no odontoblastic layer in the case of calcium hydroxide.[18] In recent times, Accorinte *et al*., have reported that pulp healing with calcium hydroxide is slower than that with MTA, when used as a pulp capping agent in human teeth.[5] Also Sarkar *et al*. have proved that MTA can bond chemically to the dentin by a diffusion-controlled reaction between the apatite layer of MTA and the dentin.[19] Of late, Chen *et al*. have shown that MTA is biocompatible and appears to have osetoconduction effects on the bone cells[20]

In our cases we used white MTA, due to esthetic concerns, in the anterior region.[21] A waiting period of 45 minutes was followed, to allow the setting of MTA, before the placement of GIC, as this was the recommended protocol by Nandini *et al*.[22] The longer setting time of MTA is a disadvantage. Of late, Kogan *et al*. have shown that NaOCl gel added as an additive has demonstrated good working times and improved setting times, but the effect of such additives on the pulp have not yet been studied.[23]

Thus, considering the age of the patient, recently traumatized teeth with pulp exposure, if treated with partial pulpotomy within 24 – 48 hours after the injury, can act as a permanent restorative procedure, without the need for endodontic treatment, provided a bacteria-tight seal is obtained.

## CONCLUSION

Thus, considering the age of the patient, recently traumatized teeth with pulp exposure in a young individual, if treated with partial pulpotomy within 24 – 48 hours after the injury, can act as a permanent restorative procedure without the need for endodontic treatment, provided a bacteria-tight seal is obtained. Further detailed clinical studies or long-term, follow-up results are needed.
